# Reviews and Book Notices

**Published:** 1881-04

**Authors:** 


### Reviews and Book Notices.

ARTICLE XI.—IMPERFECT HEARING AND THE HYGIENE OF THE EAR, INCLUDING NERVOUS SYMPTOMS, TINNITUS AURIUM, AURAL VERTIGO, DISEASES OF THE NASO-PHARYNGEAL MEMBRANE, MIDDLE EAR AND MASTOID REGION, WITH HOME INSTRUCTION OF THE DEAF. By LAURENCE TURNBULL, M.D., PH. G., etc. Third Edition, with illustrations. Philadelphia: J. P. Lippincott & Co.

In 1874 Dr. Laurence Turnbull, the well known aural surgeon of Philadelphia, published a little pamphlet on *Tinnitus Aurium.* So well was it received that in the following year a second edition had to be issued. And now the author presents to us what he calls a third edition. But title page and context have been changed so completely that we scarcely recognize the original *brochure* in this new book.

The volume opens with an excellent review of the recent progress of otology, and then discusses in a very able and entertaining manner the following subjects: 1, The limit of perception of musical tones by the human ear; 2, Tinnitus aurium; 3, Importance of treatment of the naso-pharyngeal space; 4, Artificial perforation of the membrana tympani; 5, Mastoid region and its diseases; 6, Hygiene of the apparatus of hearing, with the prevention of deafness; 7, Method of educating the deaf-mute at home; 8, Comparison between the audiphone and the various forms of ear trumpets.

A glance at this table of contents will satisfy the reader that the pages of this book are stores filled with valuable treasures; for the topics under discussion are of that high interest, scientifically and practically, that no intelligent physician can afford to be ignorant of them.

With reference to *tinnitus aurium* the author's view is defined in these remarks: "In the light of later experience, we could see no reason for changing our opinion that, in the great majority of cases, tinnitus aurium is by no means a sure indication of brain trouble, nor can it be frequently ascribed to congestion of the labyrinth, nor to auditory neuritis, except as an evidence of long continued irritation of the terminal filaments of the nerves; moreover, we had repeatedly proved that many of these cases are curable." He shows that the greater number of cases of tinnitus aurium result from some change in the external auditory meatus, membrana tympani, Eustachian tube or tympanic cavity.

In European journals we have recently read so much about *adenoid growths* in the naso-pharyngeal space that we were greatly surprised with reading this statement: "My opinion, since my return from Europe, is that the diseased condition of the post-nasal space termed 'adenoid growths' is not as frequent as it was supposed to be by Dr. Meyer, of Copenhagen, who was the first to draw attention to the disease, in a paper published in October, 1869. In the clinic of the University of Vienna, Dr. Gruber had no case on his list. I went to Paris and met Dr. Loewenberg in consultation. I most certainly thought this gentleman, who published an exhaustive treatise on the disease, would be able to show me a number of cases, but in three visits to him he was unable to show me a single case. Again when I went to Edinburgh I was most kindly invited to visit the ear clinic of the Edinburgh Royal Infirmary by the surgeon in charge, Dr. Kirk Duncanson, and was much interested in his cases, but he had no cases of this disease. In London, Dr. MacKenzie showed me every attention, and also gave me a note to his chief assistant, Dr. Mack Howell, who is medical officer and registrar of the Hospital for Diseases of the Throat and Chest, with its tens of thousands of cases, and in all their large number of instructive cases, of which I examined over one hundred, there was but one case of 'adenoid' disease in the hospital."

The chapter "The Mastoid Region and its Diseases," does not compare very well with the other parts of the book. A concise statement of the present views concerning the indications for the operations upon the mastoid would, in our opinion, be more serviceable to the reader than the histories of numerous cases.

But so much more commendable is the next chapter on the "Hygiene of the Ear." "*Of all the injurious influences combined, none, however, are so hurtful to the integrity of the human ear as cold.*" If physicians would always bear this in their mind and preach it to their patrons, and especially to the mothers and nurses of children, there would be a great deal less ear trouble; for the frequent occurrence of earache in children is usually due to the ignorant carelessness in allowing children to go out of a warm room into the cold and damp air, without sufficient wrappings.

The author, also, refers to the injurious effect of smoking, and continued sonorous vibrations, as seen in the resulting deafness of locomotive engineers, boiler makers, telegraph operators, gunners, millers, and operators in factories. And he gives a series of plain and sound directions for the care of the ear in health and disease. For these, however, we must refer the reader to the book, for the limited space of this notice forbids their reproduction in these pages.

But we cannot conclude without quoting a few lines from the last chapter; for we believe that inasmuch as all the thundering about the "audiphone" proceeded from our city, it must be highly interesting to our readers to learn a careful observer's estimate of the comparative value of the audiphone and ear-trumpets: "All are now familiar with the name of 'Audiphone,' as its virtues were heralded by the press as a great boon to the deaf, and numberless publications, lectures, etc., have been sent broadcast over the land. * * * * Our unsuccessful experiments and trials with this agent in the case of the deaf mute have been already reported and published in a lecture before the class of Jefferson Medical College. Within the last month I have taken notes of a number, all intelligent and well educated persons who have had the opportunity of testing both means of relief to their deafness, and in all but a few cases the hearing trumpet, in some form, has been preferred. The result of our experiments are as follows: In the ten cases of deafness only one was decidedly improved in the acuteness of his hearing by the audiphone, two were not benefited by either apparatus, while six were greatly improved by the various forms of the hearing-trumpet, and the one who could hear about as well with the one as with the other, preferred using the hearing-trumpet."

F. C. H.

ARTICLE XII.—A PRACTICAL TREATISE ON THE MEDICAL AND SURGICAL USES OF ELECTRICITY. INCLUDING: LOCALIZED AND GENERAL FARADIZATION; LOCALIZED AND CENTRAL GALVANIZATION; ELECTROLYSIS AND GALVANO-CAUTERY. By Geo. M. Beard, A.M., M.D., Fellow of the New York Academy of Medicine; of the New York Neurological Society, etc. A. D. Rockwell, A.M., M.D., Fellow of the New York Academy of Medicine; Electro-Therapeutist to the Woman's Hospital of the State of New York, etc. Third Edition. Revised by A. D. Rockwell, M.D. With nearly 200 Illustrations. 8vo, pp. 758. Cloth, $5.50. New York: William Wood & Co., 27 Great Jones St. 1881.

This work is above criticism. It forms a whole cyclopœdia of every principle and fact pertaining to electricity; such as its history, experiments, instruments, and medical uses. The book is divided into four parts: Electro-Physics, Electro-Physiology, Electro-Therapeutics, and Electro-Surgery. It contains a copious Glossary and an Index. But it is far above the reach of most students, and many practitioners, being particularly adapted for physicians who desire to make the treatment of diseases by electricity a specialty. The authors prove from an abundance of cases that electricity is one of the most valuable remedies, acting as a stimulant, a tonic, a sedative, or even as an anodyne, according to the manner in which it is used; and this point necessitates a great deal of knowledge and discrimination on the part of the medical attendant.

"In regard to the theory of Dr. Thomas W. Poole, of Lindsay, Canada, that electricity is essentially a paralyzing agent, and that its sedative and tonic effects are due to its paralyzing power, this may be said, that, granting for a moment the full claim, it yet remains, that practically we do obtain from the use of electricity sedative and tonic effects similar to those which we obtain from a vast number of other remedial agencies." [Extract from preface to third edition.]

A. D. ROCKWELL.

Any reader who peruses this book will be convinced that electricity is too much neglected by the profession, that it is often ill-applied, and that it is adapted to many more diseases than most of us are aware of. It is handsomely printed and illustrated. Let us hope that the authors, or some one else, will give us a compendium of the work, which would do much toward generalizing the use of electricity. H. D. V.

ARTICLE XIII.—THE PATHOLOGY, DIAGNOSIS AND TREATMENT OF DISEASES OF WOMEN, INCLUDING THE DIAGNOSIS OF PREGNANCY. By Graily Hewitt, M.D., London, F.R.C.P.; Professor of Midwifery and Diseases of Women, University College, etc. Third American, from the Third London Edition, Revised and Enlarged. With 132 Illustrations. 8vo, pp. 75. Cloth, $4.00. Philadelphia: Lindsay & Blakiston. 1880.

"This New Edition of my Treatise on 'Diseases of Women' is substantially a new work." Preface to the Third Edition.

The expectations of students and practitioners will be liberally rewarded by a careful perusal of this book. The treatment is specially commendable, and will be found practicable by most physicians. Dr. Hewitt's views are broad, and he takes into consideration the results obtained by all the famous specialists now engaged in the treatment of female diseases, in England as well as in America. He has not the originality of Emmet, nor that of Thomas, but for most students this might as well be considered his chief advantage. Too much space consecrated to explain and exemplify one's peculiar views, is not desirable in medicine, which derives its value from so many sources. The chapter on the Diagnosis of Pregnancy is thorough and valuable. It is very appropriate in a work like this, especially since an operator two years ago in this country, made a Cæsarean section on a girl, pregnant for some months, with the intention of removing a tumor.

On page 454, line 25, we read: "In the vaginal mucus Donné found, on examination by the microscope, a number of *trichomonata*, which are oval, shaped like a pear or biscuit, and are from six lines to an inch and four lines long." This is a pretty large animalcule. I wonder why "Beigel has failed in finding them," as we read five lines below. The book is published in good style

H. D. V.

ARTICLE XIV.—THE RELATIONS OF GOITRE TO PREGNANCY AND DERANGEMENTS OF THE GENERATIVE ORGANS OF WOMEN. By Edward W. Jenks, M.D., LL.D., Chicago, Ill.

This is a very interesting paper of thirty-nine pages, reprinted from the *American Journal of Obstetrics*, etc., for January, 1881.

The author, after a thorough examination of the literature of the profession touching the subject of goitre, has brought together in these pages a full and instructive résumé of what has been observed concerning the relations of goitrous swellings and morbid growths to functional activity and derangements of the female organs of generation.

The author has also given due attention to the treatment of the various forms of trouble under consideration. His conclusions are as follows:

"CONCLUSIONS.

"1st. There is indisputable evidence that there may be endemic and occasionally epidemic causes producing goitre in men as well as women, yet the evidence is equally indisputable that every form of goitre occurs among the latter in a much larger proportion than among the former.

"2d. The fact has long been established that in certain occult conditions of women, increased vascularity and enlargement of the thyroid gland may be produced as a consequence of some unusual excitement of the generative organs.

"3d. Violent parturient efforts may cause the vascular form of goitre, but under the influence of pregnancy there may be gradual enlargement of the thyroid gland lasting for years; while, on the contrary, a goitre produced by one pregnancy is sometimes cured by a subsequent one.

"4th. There are reasons for believing that, when goitre is caused by any disorder of the generative organs (excepting pregnancy), it is due more commonly to functional than to structural disease.

"5th. It is not as a consequence of phlegmasias, or malignant diseases of the uterus or its annexes, that goitre is develope l; on the contrary, the disorders which more commonly cause or precede goitre are fluxions, congestions, functional diseases of the pelvic organs, or those disorders of menstruation which are of systemic origi .

"6th. As many goitrous necks among women are due solely to some derangement of their generative organ-, the use of topical applications or remedies, however administered, unless made use of to remedy the cause, will be of no avail, and constitutes irrational and unscientific treatment.

"7th. In the prognosis of goitre, we should always bear in mind the possible complications when the tumor is of considerable size; prominent among which are compression of the trachea, leading to dyspnœa, or even dysphagia, and compression of the recurrent laryngeal nerve, producing harshness of the voice, and sometimes aphonia.

"8th. When the goitre is not large and is manifestly dependent upon some derangement of menstruation, some functional uterine affection, or has suddenly developed in consequence of pregnancy or violent efforts in labor, the prognosis is fav rable, although it is not certain that there will be a rapid disappearance of the deformity."

---

HYDROBROMATE OF MORPHIA.—Dr. Landrieux (*La France Médicale*) employs this salt in solution to 1-50th, and injects from 5 milligrammes to 1 and 2 centigrammes a day. More soluble than the hydrochlorate of morphia, it may be used in half doses, is more sedative, and does not give rise to the sort of purulent diathesis, frequent after repeated injections of hydrochlorate.

